# Insights into the Implementation of Partial Denitrification-Anammox in Actual Municipal Wastewater: A Comparison of Two Strategies

**DOI:** 10.1021/acsomega.5c11568

**Published:** 2026-04-24

**Authors:** Paula Yumi Takeda, Carolina Tavares Paula, André do Vale Borges, Julio Cesar Pereira Cantarelli, Elis Watanabe Nogueira, Maria Eduarda Simões Dias, Márcia Helena Rissato Zamariolli Damianovic

**Affiliations:** Biological Processes Laboratory (LPB), São Carlos School of Engineering (EESC), 67817 University of São Paulo (USP), Av. João Dagnone, 1100, Santa Angelina, São Carlos, São Paulo 13563-120, Brazil

## Abstract

Efficient and sustainable nitrogen removal is vital for wastewater treatment, and the partial denitrification-anammox (PDA) process shows promise for achieving high performance. The responses of anammox to PDA implementation with different strategies (gradual and abrupt bioprocess integration) were evaluated. Anammox (AnAOB) enrichment was initially achieved under synthetic medium conditions with a nitrogen removal efficiency (NRE) of 85.5%, which increased to 94.3% after the introduction of real wastewater, demonstrating system robustness and adaptability. The gradual PDA implementation strategy involved controlled nitrate supplementation and progressive adjustment of nitrate–nitrite availability, promoting cooperative interactions between AnAOB and denitrifying bacteria (DNB), resulting in 75.4 ± 5.0%, with heterotrophic denitrification contributing up to 57.4% of NRE. In contrast, abrupt PDA implementation relied on direct high-nitrate exposure, leading to distinct nitrogen conversion pathways, with an anammox reaction contribution of 58.1 ± 2.8%, although with low ammonium removal of 33.1 ± 0.8%. The availability of refractory organic compounds from the anaerobically pretreated municipal wastewater was identified as the limiting factor for higher substrate production (nitrite) levels for AnAOB during partial denitrification. A pronounced shift in microbial diversity was observed, with a notable increase in the abundance of partial denitrifiers from the *Thauera* genus, rising from 1.2 to 28.2%, in response to the abrupt PDA implementation. The present study provides insights into the successful implementation of PDA treating actual wastewater under mainstream conditions.

## Graphical abstract

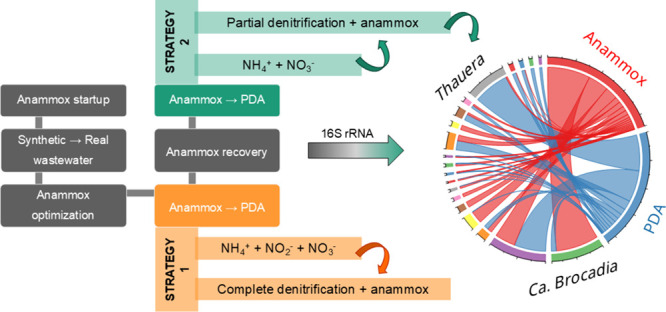

## 1 Introduction

As environmental regulations for wastewater discharge become increasingly stringent, there is a pressing need for more efficient and sustainable treatment technologies.^1^ Conventional nitrification and denitrification processes, while effective, are often costly and energy-intensive. This has driven interest in alternative methods that offer enhanced performance and reduced operational costs.^2^ The partial denitrification-anammox (PDA) process has emerged as a promising alternative due to its potential for high efficiency and reduced operational costs, presenting a more sustainable solution for nitrogen removal.^2^ Compared to the widely studied partial nitrification-anammox process, PDA is considered more stable,^3^ and has recently gained increased attention.

Despite its advantages, initiating the PDA process remains a significant challenge. Start-up strategies may involve using only anammox sludge or combining it with anaerobic or denitrifying sludge.^4,5^ Effectively managing this step is crucial for establishing a balanced consortium of anammox bacteria (AnAOB) and denitrifying bacteria (DNB) and ensuring stable process performance. Traditionally, the start-up is performed in synthetic medium, which limits process scalability and masks challenges associated with real wastewater complexity. Furthermore, the transition from synthetic to actual municipal wastewater represents a critical but underexplored bottleneck for PDA implementation, particularly regarding microbial community stability and nitrogen conversion pathways.^4,6^

The municipal wastewater contains complex organic matter, a considerable proportion of which is removed during pretreatment, leaving less suitable organic carbon for DNB involved in the PDA process.^7^ This can impact nitrogen removal efficiency (NRE) and microbial activity, limiting the denitrification pathway.^8^ Additionally, nitrate concentrations in anaerobically pretreated municipal wastewater (APMW) are insufficient for effective PDA operation, necessitating optimization of the carbon-to-nitrate and carbon-to-nitrogen ratio (C/N).^9,10^ Research highlights the importance of appropriate carbon sources for heterotrophic denitrification (HD) and the need to control nitrate to nitrite conversion to maintain PDA stability.^3,8^ In this context, the establishment of PDA critically depends on how nitrate availability and nitrite supplementation are managed, which can be achieved either through a gradual adjustment or an abrupt shift in nitrate–nitrite profiles. While acetate has shown promise as a carbon source,^4,6^ using actual APMW to provide organic matter could be a cost-effective alternative for PDA.^2^ However, comparative evaluations of PDA implementation strategies (abrupt and gradual) under low-strength municipal wastewater remain scarce, and reports on the performance of PDA during the transition from synthetic to real wastewater are still limited.

In this scenario, the present study aims to directly compare gradual PDA (gradual nitrate supplementation followed by nitrite reduction) and abrupt PDA (direct high-nitrate implementation) integration strategies during the transition from synthetic to actual municipal wastewater, focusing on AnAOB–DNB consortium establishment, nitrite control, and process stability. This research investigated the practical application of AnAOB technology with the objective of contributing to the development of efficient and sustainable wastewater treatment solutions.

## 2 Materials and Methods

### 2.1 Bioreactor Setup and Operation

A laboratory-scale upflow reactor (0.7 L) was applied to investigate the nitrogen removal of mainstream municipal wastewater at 37 °C, maintaining a short hydraulic retention time (HRT) of 2 h (Figure 1). The bioreactor was inoculated with active granular anammox biomass from a prior study by Takeda et al.^11^ at a concentration of 3.5 g L^–1^, in terms of volatile suspended solids. Its operation was divided into six phases, during which anammox and PDA processes were investigated, as well as the impact of synthetic and actual wastewater, as detailed in Table 1. First, during phase 1, synthetic medium (SM) was carried out based on van de Graaf et al.^12^ The substrates for the anammox reaction (N-NH_4_^+^ and N-NO_2_^–^) were provided by adding (NH_4_)_2_SO_4_ and NaNO_2_, respectively, under an influent substrate ratio of 1 N-NH_4_^+^:1.32 N-NO_2_^–^. Prior to the feeding, the SM was purged with a gas mixture of argon (95%) and carbon dioxide (5%) to reduce the dissolved oxygen (DO) concentration below 0.15 mg L^–1^.

**Figure 1:**
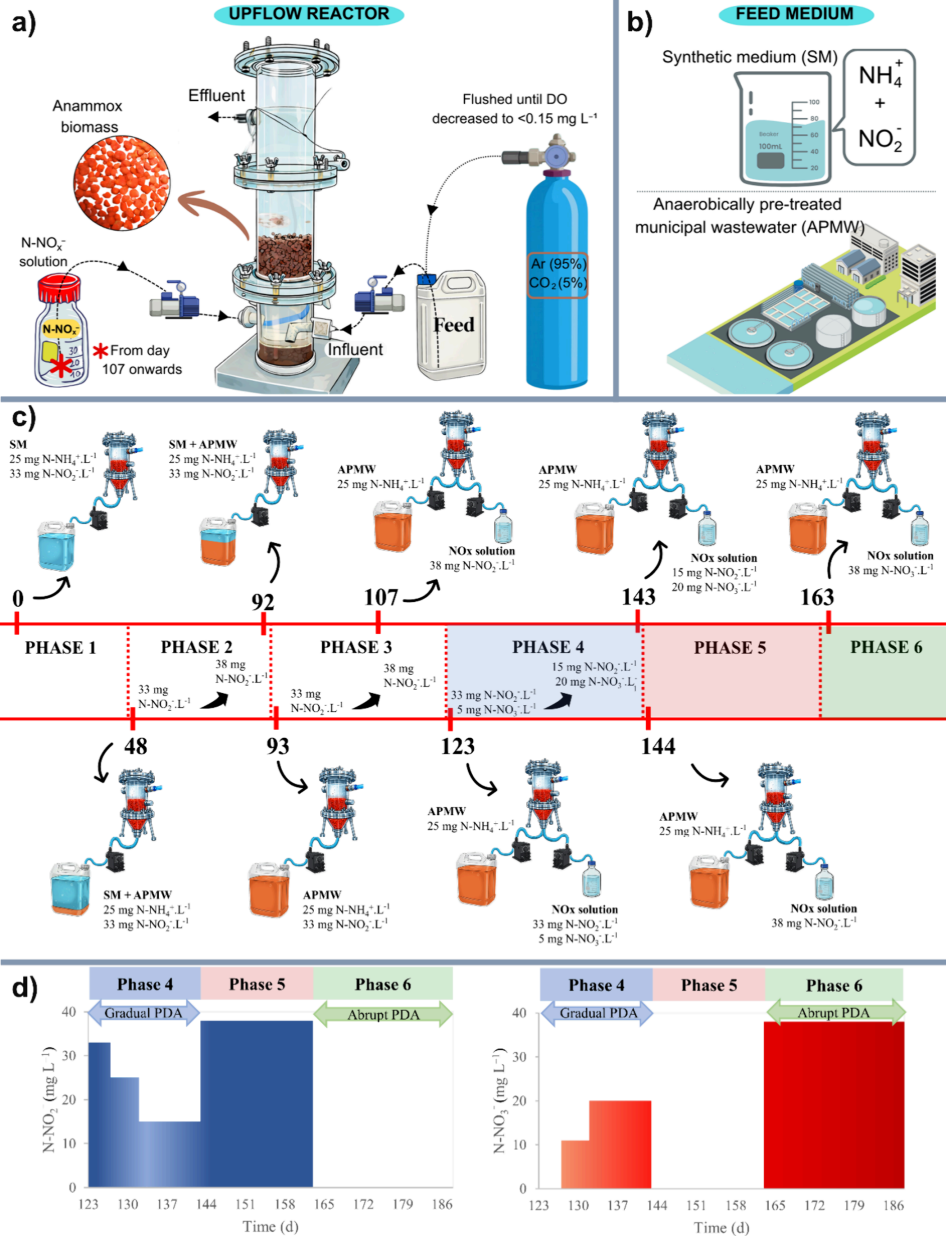
Schematic representation of the operational phases: (a) laboratory-scale upflow bioreactor configuration; (b) composition of the feed medium; (c) schematic illustration of the transition from synthetic medium (SM) to anaerobically pretreated municipal wastewater (APMW), including supplementation with N–NH_4_^+^, N–NO_2_^–^, and N–NO_3_^–^; and (d) conceptual representation of the gradual and abrupt implementation strategies of the partial denitrification–anammox (PDA) process.

**Table 1:** Operating Conditions Applied to the PDA Reactor^a^

phase	operation time (d)	wastewater	main process	N-NH_4_^+^ (mg L^–1^)	N-NO_2_^–^ (mg L^–1^)	N-NO_3_^–^ (mg L^–1^)
1	1–47	SM	AMX	25	33	0
2	48–92	SM + APMW	AMX	25	33	0
3	93–122	APMW	AMX	25	33 → 38	0
4	123–143	APMW	PDA	25	33 → 15	5 → 20
5	144–162	APMW	AMX	25	38	0
6	163–188	APMW	PDA	25	0	38

a SM: synthetic medium; APMW: anaerobically pretreated municipal wastewater; AMX: anammox process; PDA: partial denitrification-anammox.

Afterward, in phase 2, APMW from the treatment plant in São Carlos (SP, Brazil) was added to the SM. The proportion of APMW in the mixture was gradually increased until the reactor ended up being exclusively fed with APMW from phase 3 onward (Figure 2). The physicochemical characterization of the collected APMW counted with five repetitions and it was conducted following the procedures outlined in the Standard Methods for the Examination of Water and Wastewater.^13^ The measured physicochemical properties of the APMW were: chemical oxygen demand (COD; 56.3 ± 4.9 mg L^–1^); pH (7.2 ± 0.1); ammonium (N-NH_4_^+^; 23.6 ± 1.6 mg L^–1^); nitrate (N-NO_3_^–^; 1.3 ± 0.2 mg L^–1^); total alkalinity (185 ± 12 mgCaCO_3_ L^–1^); total suspended solids (TSS; 130 ± 17 mg L^–1^) and volatile suspended solids (VSS; 71 ± 8 mg L^–1^).

**Figure 2:**
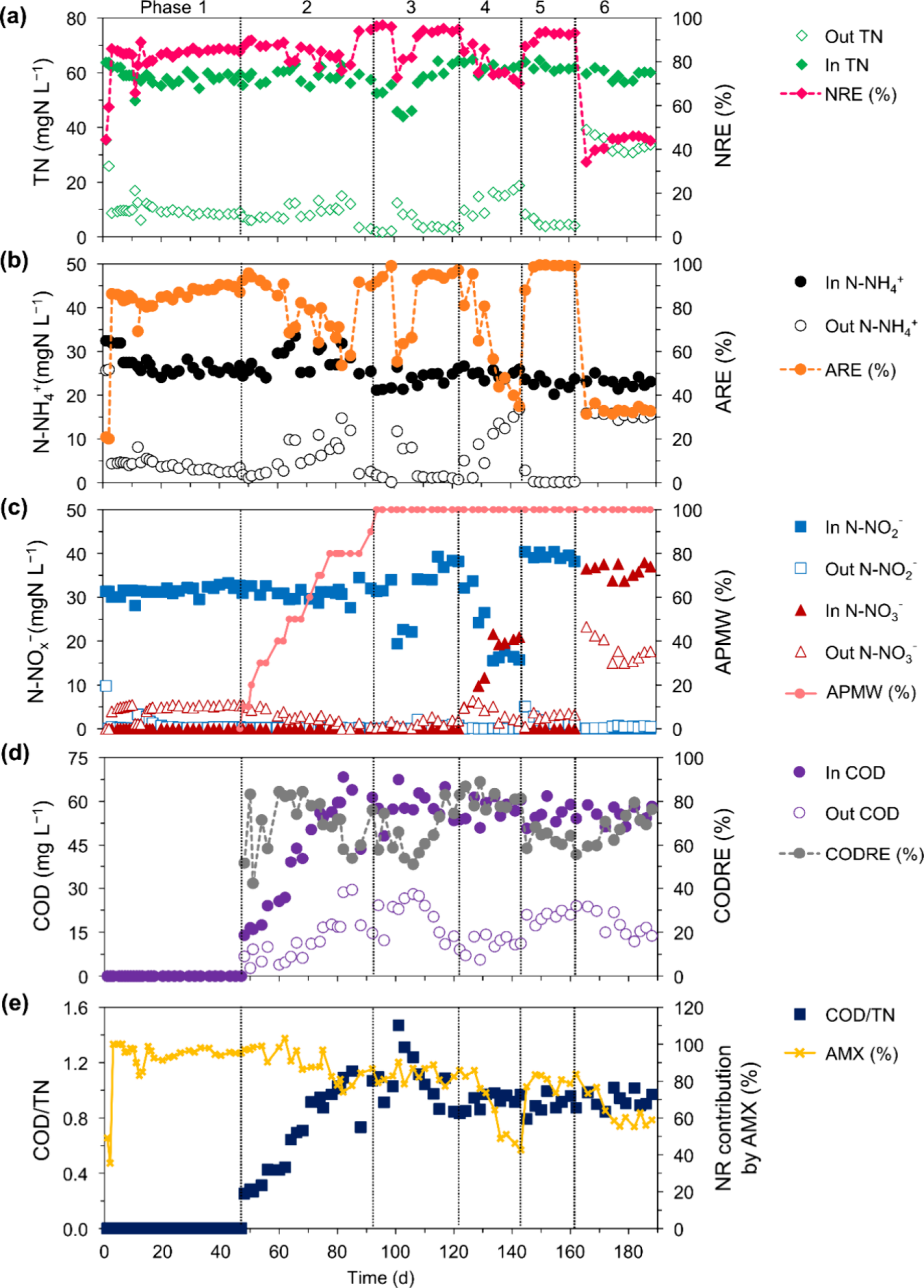
Temporal profiles: (a) Total nitrogen (TN) concentration, nitrogen removal efficiency (NRE); (b) N-NH_4_^+^ concentration, ammonium removal efficiency (ARE); (c) N-NO_2_^–^, N-NO_3_^*‑*^ concentration, anaerobically pretreated municipal wastewater (APMW) (%); (d) chemical oxygen demand (COD), organic matter removal efficiency (CODRE); (e) COD/N ratio, Nitrogen removal (NR) contribution by anammox (AMX) (%).

During phases 3 and 4, influent concentrations of nitrite and the ratio of N-NH_4_^+^:N-NO_2_^–^ varied (Figures 1 and 2). The COD/N ratio of the APMW was controlled through batch-wise adjustment prior to reactor feeding by measuring COD, N-NH_4_^+^, and N-NO_3_^–^ concentrations. Based on these measurements, the required amount of N-NO_2_^–^ was calculated and externally added to the influent in order to adjust the total nitrogen concentration and maintain a target COD/N ratio of 0.9. Subsequently, N-NO_3_^–^ was supplied in the form of NaNO_3_ in phases 4 and 6 to investigate the PDA process. This approach enabled the examination of different PDA implementation strategies for gradual and abrupt increase in such compound, respectively_._ Phase 5 was performed to recuperate the anammox activity in the reactor, with N-NO_2_^–^ provided instead of N-NO_3_^–^ (Figure 2). Throughout the experimental period, nitrogen loading rate was maintained approximately at 0.7 gN L^–1^ d^–1^. Consequently, the concentrations of oxidized N-compounds were adjusted to support this loading rate.

During the initial experimental phases until day 106 (phases 1–3), nitrogen compounds were supplemented directly in the feed tank. On the other hand, from day 107 onward (phases 3–6), nitrogen compounds were provided from a separated tank, prepared at a higher concentration of 10 gN-NO_*x*_^–^ L^–1^, which was continuously dosed into the influent line using a peristaltic pump at a low flow rate, synchronized with the main feed, in order to avoid wastewater dilution and localized substrate concentration peaks (Figure 1). Prior to the feeding, this solution was purged with the previous gas mixture (Ar/CO_2_), lowering the DO concentration below 0.15 mg L^–1^.

### 2.2 Analytical Methods and Calculations

The concentrations of N-NH_4_^+^, N-NO_2_^–^, N-NO_3_^–^, COD, and pH were determined following the protocols described in the Standard Methods for the Examination of Water and Wastewater.^13^ The N_2_O production was assessed with a gas chromatograph, as described by Pereira et al.^14^ The nitrogen mass balance was calculated as described by Pijuan et al.^15^

### 2.3 Ex Situ Specific Microbial Activity

Specific anammox activities tests (SAA) and specific heterotrophic denitrification activities tests (SHDA) were conducted in 0.5 L flasks at the end experimental phases 1, 3, and 6. Granular biomass was washed with mixed liquor containing trace elements^12^ to eliminate residual inorganic nitrogen and organic carbon being added to the corresponding flasks. To create an anaerobic/anoxic environment in each flask, before introducing the respective substrates, the solution was purged with Ar/CO_2_ for 10 min coupling a porous stone at the end of the air pipeline for better diffusion.

For the SAA tests, 25 mgN-NH_4_^+^ L^–1^ and 33 mgN-NO_2_^–^ L^–1^ were added; and for SHDA tests, 20 mg NO_3_^–^-N L^–1^ and 100 mg COD L^–1^ (potassium acetate). The purged flasks were then fixed and incubated at 130 rpm and 37 °C. Liquid samples were taken with syringes at regular intervals over a maximum period of 12 h for analysis of the N-compounds and COD. Further methodological details can be found in Takeda et al.^11^

### 2.4 Microbial Community Analysis

Two samples were collected for 16S rRNA gene amplicon sequencing analysis at the end of experimental phases 3 (sample P3) and 6 (sample P6). The microbial community analysis was carried out as described by Takeda et al.^11^ using the 515FB/806RB set of primers. The data sequences are available in the National Center for Biotechnology Information (NCBI) database through the project access number PRJNA1135914.

## 3 Results and Discussion

### 3.1 Anammox Domestication from Synthetic to Actual Wastewater: Phases 1 to 3

Figures 2 and 3 illustrate the reactor performance throughout the experimental period, and the microbial specific activity, respectively. Under optimal conditions for AnAOB enrichment in phase 1, a high NRE of 85.5 ± 0.5% was achieved (Figure 2a), which is close to the maximum theoretical value of 89%, taking into account the nitrate produced during the anammox reaction. Furthermore, the average removed nitrite to removed ammonium (N-NO_2_^–^:N-NH_4_^+^) and produced nitrate to removed ammonium (N-NO_3_^–^:N-NH_4_^+^) ratios were of 1.39 ± 0.03 and 0.23 ± 0.01, respectively. These values are close to the theoretical stoichiometric ratios proposed by Strous et al.^16^ for the anammox reaction, which are 1.32 N-NO_2_^–^:1 N-NH_4_^+^ and 0.26 N-NO_3_^–^:1 N-NH_4_^+^. Additionally, the SAA test showed higher anammox activity of 31.7 mgN gVSS^–1^ d^–1^ (Figure 3), compared to the seeding sludge activity of 28.2 mgN gVSS^–1^ d^–1^.^11^

**Figure 3:**
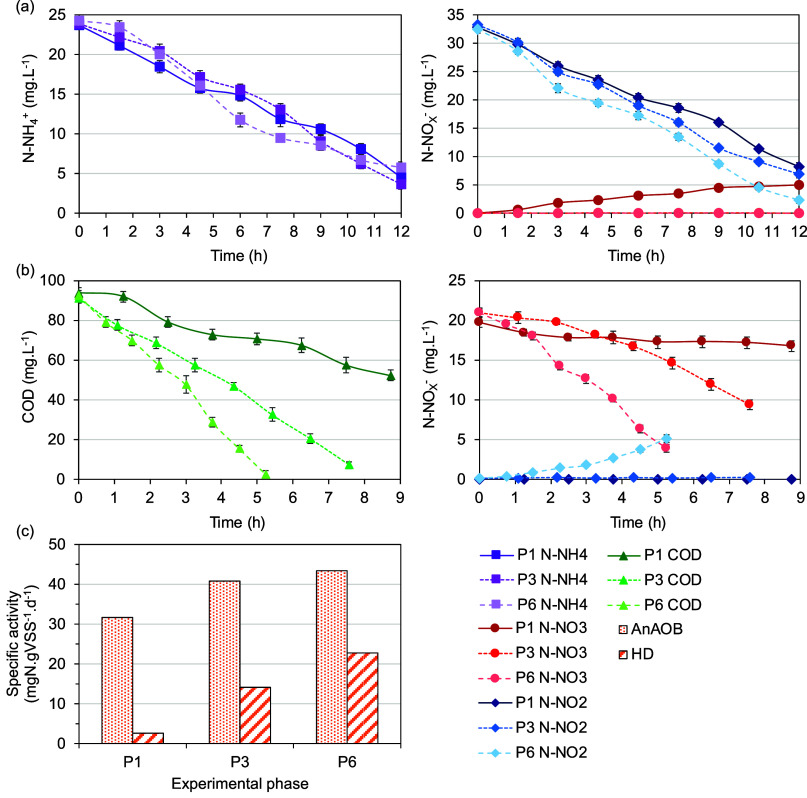
Specific microbial activity: (a) SAA temporal profiles; (b) SHDA temporal profiles; (c) specific activity.

Domestication was initiated from day 48 onward in phase 2, during which APMW was gradually added to the SM in varied proportions, as shown in Figure 2c. Microbial community adaptation to APMW lasted 44 days. During this period, increasing influent concentration of organic matter, up to 68 mg COD L^–1^, established conditions for HD. While HD can be beneficial to NRE by reducing the nitrate produced by AnAOB to nitrite,^2^ thereby providing a substrate for the anammox reaction, an increased competition for nitrite was expected. As low SHDA was obtained at the end of phase 1 (2.6 mgN gVSS^–1^ d^–1^), HD accounted for a minority of the nitrogen removal (19.8 ± 4.3%), with the anammox pathway responsible for about 80.2 ± 4.3% (Figure 2e). The higher AnAOB contribution for NRE is corroborated by the high ammonium removal efficiency (ARE) of up to 91.6% (Figure 2b) at an APMW:SM ratio of 90:10 (v/v). As expected in simultaneous anammox-HD process, a low effluent nitrate concentration of 0.8 ± 0.7 mgN-NO_3_^–^ L^–1^ was obtained (Figure 2c). This domestication stage to actual wastewater was essential to ensure high AnAOB activity and competitiveness.

Finally, in phase 3, during which the reactor was fed solely with APMW, improved ARE and NRE were observed of 95.2 ± 1.1 and 94.3 ± 0.6%, respectively. Despite the low growth rate of AnAOB, its SAA increased to 40.8 mgN gVSS^–1^ d^–1^, which is consistent with the higher ARE observed compared to phase 1. Regarding nitrogen removal, HD played a significant role in increasing NRE, as well as improving the organic matter removal efficiency, in terms of COD (CODRE), achieving 73.1 ± 8.4%. This high reactor performance most likely resulted from two approaches: the previous domestication stage of gradually introducing actual wastewater, and the increased influent nitrite supply for the anammox pathway (Figure 2c). Furthermore, no anammox reaction inhibition was observed at organic matter concentrations up to 67.4 mg COD L^–1^.

On the 101st day, nitrite supply in the feed tank was consumed, possibly by the DNB present in the APMW, resulting in an influent concentration of approximately 20 mgN-NO_2_^–^ L^–1^. As expected, under this influent condition, a low ARE of 55.3% was obtained, since insufficient nitrite was available for the anammox reaction. Therefore, from day 107 onward, nitrite was supplied in a separate tank from the APMW. This approach ensured the nitrite influent concentration required for the stoichiometric anammox reaction of 1.32 N-NO_2_^–^:1 N-NH_4_^+^.^16^ However, maintaining the influent ratio of 1.32 N-NO_2_^–^:1 N-NH_4_^+^ was still insufficient to sustain high ARE, as DNB significantly increased their activity, competing with AnAOB for nitrite. In fact, SHDA increased 7-fold compared to phase 1, reaching 14.1 mgN gVSS^–1^ d^–1^. For that reason, the influent N-NO_2_^–^:N-NH_4_^+^ ratio was increased to 1.5. Similarly, Fernandes et al.^17^ and Leal et al.^18^ reported ratios up to 2.0 to meet the demands of both microbial communities. Furthermore, Fernandes et al.^17^ highlighted the feasibility of implementing the anammox process for treating APMW.

It is noteworthy to mention that ensuring AnAOB retention is crucial for successful reactor operation due to its lower growth rate compared to HD bacteria.^2,8^ Supplying organic matter to the reactor may lead to increased development of heterotrophic microorganisms, potentially outcompeting other autotrophic microorganisms. Besides competing for nitrite, DNB also compete with AnAOB for space.^19^ Therefore, controlling the influent C/N ratio is essential to limit the growth of heterotrophic microorganisms and prevent inhibition of autotrophic ones. Municipal wastewater commonly contains C/N ratios ranging from 7 to 12,^20^ which is higher than the values typically recommended for anammox-based reactors (1–3).^2,19^ Lowering this ratio is crucial to achieve a stable mainstream anammox process. In this study, feeding the reactor with APMW in phase 3 was essential for applying a reduced influent COD/N ratio of 0.9 ± 0.1. This ratio created a synergistic environment for both the denitrifiers community, autotrophics and heterotrophics, as evidenced by the increased AnAOB contribution to NRE of 83.6 ± 3.9% and high CODRE (73.1 ± 8.4%).

### 3.2 Gradual PDA Implementation: Phase 4

In the first operational strategy to develop a single-stage PDA system, oxidated N-compounds varied in phase 4 by decreasing influent nitrite concentration and increasing nitrate concentration, as shown in Figure 2c. A stable nitrite supply is essential as a substrate for the anammox reaction to enhance nitrogen removal through its pathway. This strategy resulted in an ARE of 50.7 ± 15.0%, while maintaining an influent nitrite concentration of approximately 16 mgN-NO_2_^–^ L^–1^ (Figure 2b,c). However, NRE decreased significantly (*p* < 0.05) to 75.4 ± 5.0% (Figure 2a).

At average COD/N ratio of 0.95 ± 0.02, consistent to the previous phase 3, CODRE increased to 79.5 ± 2.7% (Figure 2d), indicating greater heterotrophic activity. As expected, a rising trend in HD contribution to nitrogen removal was observed, reaching up to 57.4% on day 143 (Figure 2e). Concurrently, the decline in ARE activity indicates that denitrification has surpassed the anammox process. Single-stage PDA systems have been reported to achieve NRE higher than 85% when treating mainstream synthetic wastewater with easily biodegradable organic matter, such as acetate.^21,22^ In contrast, lower efficiencies (<80%) have been observed when applying actual municipal wastewater,^5,23^ possibly due to a more complex and refractory carbon sources found in this effluent, as in the present study. Additionally, these authors employed a higher C/N ratio of 3.0 and obtained nitrogen removals by anammox reaction of 45 ^5^ and 69%.^23^ Despite the lower organic matter levels in the present study, the gradual transition strategy resulted in low nitrogen removal via the anammox pathway. Therefore, nitrite influent supply was possibly reduced to N_2_ by HD bacteria, surpassing AnAOB.

According to Yang et al.,^23^ nitrite supplementation is effective at C/N ratios as low as 2.5, but at ratios as high as 3.5, it enhances complete denitrification over the anammox process. However, in the present study, with a lower COD/N ratio (0.96 ± 0.04), nitrite supplementation most favored complete denitrification. Several operational factors may explain this difference. Yang et al.^23^ operated the reactor at temperatures ranging from 15 to 28 °C, conditions that favor controlled nitrite accumulation and selective enrichment of partial denitrifiers. In contrast, the present study treated APMW, which is characterized by low concentrations of readily biodegradable organic matter and a predominance of refractory compounds. Under these conditions, the limited but continuously available fraction of slowly biodegradable carbon may have sustained heterotrophic activity over time, promoting complete nitrite reduction despite the low bulk COD/N ratio.

Furthermore, differences in HRT may have influenced nitrite availability and microbial competition dynamics, as longer contact times can facilitate further nitrite reduction by HD. Therefore, while Yang et al.^23^ demonstrated a C/N-dependent regulation of nitrite accumulation under moderate temperatures and mainstream conditions, the present findings suggest that, when treating complex wastewater at higher temperature, substrate quality and operational parameters may outweigh the influence of the bulk COD/N ratio on the balance between partial and complete denitrification.

Temperature was maintained at 37 °C to enhance AnAOB growth.^24^ Anammox bacteria exhibit strong temperature dependence, with sharp declines in growth rate and specific activity below 20 °C, longer start-up periods, and higher susceptibility to process disturbances; maintaining sufficient anammox biomass and stable N removal at 10–15 °C is therefore considerably more difficult than under mesophilic conditions.^25–28^ Nevertheless, several lab, pilot and full-scale studies have shown that with long-term acclimation and appropriate reactor configuration, anammox activity and abundance can be sustained or even increase as temperature decreases into the mainstream range, and high N removal can still be achieved.^5,29–33^ Lower temperatures also alter the balance between PD and anammox by differentially affecting the key functional bacterial groups. Recent studies have indicated lower nitrite accumulation by PD at higher temperatures (30 °C).^5,22^ According to Liu et al.,^22^ at lower temperatures (20 °C), the ratio of nitrite supply to consumption through the HD pathway increases, as it decreases the nitrite reduction to N_2_. Therefore, coupling PD to anammox at 37 °C potentially resulted in complete HD of the oxidized N-compounds, corroborating the data obtained of 57.4% nitrogen removal by HD. It is noteworthy that no denitrifying sludge was inoculated in the reactor, suggesting that denitrifiers proliferated as a consequence of the operational conditions (substrate availability, organic carbon source, temperature), as well as from the wastewater.^34^

In PDA systems, cooling from 20–30 to 10–15 °C can enhance partial denitrification or denitratation, increasing nitrite availability and, in some cases, the relative contribution of anammox to N removal despite reduced specific anammox activity.^5,29,31,35,36^ Heterotrophic denitrifiers such as *Thauera* spp. and related PD bacteria (e.g., Bacillus, Dechloromonas, Ferribacterium) are frequently enriched and remain highly active under ambient conditions, where they serve as important nitrite suppliers to anammox but may also compete for nitrite or drive full denitrification if not properly controlled.^21,35–39^ Furthermore, temperature-driven changes in hydrolysis, fermentation and electron-donor availability can modulate the relative contributions of PD and anammox and influence the selection of PD communities.^21,31,37,38^ Together, these effects may reduce the resilience of the coupled PDA process to load and dissolved oxygen fluctuations and increase the risk of nitrite accumulation or loss of anammox activity under mainstream conditions, particularly without adequate biomass retention and nitrite control.^5,25,26,31,32,35,40^

### 3.3 Anammox Activity Recovery: Phase 5

Due to the low NRE and to prevent further deterioration of the anammox reaction from the previous experimental phase, nitrite supply for AnAOB returned to being fully provided by chemical addition starting from day 144 onward. Under this condition, rapid recovery of the anammox reaction was observed, achieving an ARE of 88% on day 145 (Figure 2b). Furthermore, the anammox pathway was responsible for an average of 80.1 ± 3.4% NRE (Figure 2c). Concurrently, HD activity decreased as CODRE was reduced to approximately 62.0 ± 3.4%. Recent studies have reported the recovery of anammox activity by eliminating influent organic matter supplementation, achieving a null value for the C/N ratio, with recovery taking at least 17 d.^41,42^ In contrast, in the present study, faster recovery was achieved while still maintaining a COD/N ratio of 0.91 ± 0.05. Shortly, the approach implemented may represent a more feasible strategy for rapid recovery of anammox activity.

### 3.4 Abrupt PDA Implementation: Phase 6

In experimental phase 6, nitrite supply was abruptly shifted to nitrate (Figure 2c), as a different strategy to achieve PDA, compared to phase 4. Influent organic matter concentrations slightly decreased to 55.2 ± 2.5 mg COD L^–1^ compared to previous phases, but without significant difference (*p* > 0.05), resulting in an average COD/N of 0.94 ± 0.04, although APMW commonly presents greater ratios of 2–7.^7^ The presence of this influent condition, in conjunction with higher nitrate influent levels, was sufficient to reduce nitrate denitratation by approximately 54.2 ± 1.4%. Consequently, a lower substrate (nitrite) was available for AnAOB, resulting in a low ARE of 33.1 ± 0.8%, compared to phase 4. Furthermore, despite the low COD/N ratio, the growth of other heterotrophic microorganisms, nondenitrifiers, such as the hydrolytic and acidogenic microbes, may have been the main cause of this limited nitrate reduction. Nevertheless, anammox pathway was responsible for 58.1 ± 2.8% of NRE. Similar reports have shown 51% NRE via anammox at a C/N ratio of 0.9.^42^ The marked increase in the anammox pathway’s contribution to nitrogen removal in this phase, as compared to phase 4, might be associated with the implemented strategy of abrupt exchange in N-NO_*x*_^–^ supply. This finding suggests a predominance of partial denitrification over complete denitrification. Additionally, these results indicate alterations in the nitrogen removal dynamics by modifying the influent N-NO_2_^–^: N-NO_3_^–^ ratio for more refractory C-sources wastewater.

As highlighted in Section 3.2, the complexity of organic matter in APMW likely played a key role in being responsible for the low nitrate reduction, since the biodegradable fraction of municipal wastewater is mostly removed during anaerobic treatment. Several studies have utilized acetate as an exogenous carbon source to enhance the ratio of nitrate transformation to nitrite and to enrich microorganisms responsible for PD.^4,43,44^ Additionally, these studies have proposed a C/N ratio range of 1.5–3.0 for achieving high nitrite accumulation, with reported ratios as high as 97%. Both factors, complexity of carbon source and its low concentration, potentially contributed to the limited denitratation and consequently to reduced anammox reaction rates. In accordance, Yang et al.^45^ observed elevated PD activity in an anammox reactor fed with effluent from a partial nitration reactor, containing refractory organic compounds, and readily biodegradable carbon source (acetate), at a low COD/N ratio (0.9), in comparison to effluent from a partial nitration reactor alone. Therefore, for engineering applications, it is recommended to bypass raw municipal wastewater while controlling the appropriate COD/N ratio and ensuring sufficient biodegradable organic matter supply.

Recent rapid start-up strategies for partial nitrification-anammox (PN/A) and PDA systems have demonstrated high nitrogen removal efficiencies under optimized and controlled conditions. Yu et al.^46^ achieved a rapid transition from complete nitrification to PN/A at 15 °C by selectively inactivating nitrite oxidoreductase, treating synthetic wastewater containing approximately 300 mg N-NH_4_^+^ L^–1^. Their strategy resulted in total nitrogen removal efficiencies above 85–87% within a short operational period, mainly due to effective nitrite oxidizing bacteria suppression and stable nitrite accumulation. In contrast, the present study operated at 37 °C using anaerobically pretreated municipal wastewater with much lower nitrogen concentrations and a low COD/N ratio (0.9), without targeted enzymatic suppression. Under the gradual PDA strategy (phase 4), ARE reached 50.7 ± 15.0%, while NRE decreased to 75.4 ± 5.0%, mainly due to heterotrophic denitrification contributing up to 57.4% of nitrogen removal. Therefore, unlike Yu et al.,^46^ where nitrite oxidizing bacteria control was the key limiting factor, in the present system the main limitation was competition for nitrite between AnAOB and heterotrophic denitrifiers under carbon-restricted and refractory conditions.

Similarly, Hu et al.^6^ reported rapid start-up of partial denitrification coupled with anammox using innovative operational controls and readily biodegradable carbon sources. Operating at optimized COD/N ratios of 3.0, they achieved nitrite accumulation ratios exceeding 85–90% and stable TN removal efficiencies above 80–85%. In contrast, the present study operated at a significantly lower COD/N ratio (∼0.94 to 0.96), without external acetate addition. During the abrupt PDA implementation (phase 6), nitrate reduction reached 54.2 ± 1.4%, and the anammox pathway contributed 58.1 ± 2.8% to NRE, although overall ARE decreased to 33.1 ± 0.8%. Notably, despite carbon limitation, AnAOB consumed approximately 49.4 ± 3.6% of the nitrite generated, demonstrating strong competitiveness for nitrite even under low biodegradable COD availability. While Yu et al.^46^ and Hu et al.^6^ achieved rapid start-up and high efficiencies under synthetic or carbon-optimized conditions, the present work demonstrates PDA implementation under real APMW with refractory organic matter and low COD/N.

Some studies have investigated the optimal HRT for PD, where nitrite concentrations reach their peak. HRTs varying from 0.2 to 1.0 h have been implemented and reported to achieve a nitrate-to-nitrite transformation ratio greater than 85% when treating municipal wastewater.^47,48^ Therefore, longer reaction times would be necessary for the degradation of residual recalcitrant compounds present in APMW to, then, generate nitrite, which could further be consumed in the anammox reaction. This could explain the observed low nitrate reduction and ammonium removal at an HRT of 2 h. Nevertheless, considering the stoichiometry of the anammox reaction (1.32 N-NO_2_^–^:1 N-NH_4_^+^),^16^ AnAOB consumed approximately 49.4 ± 3.6% of the nitrite generated through denitratation. This result highlighted the great AnAOB competitiveness for nitrite. Therefore, the levels of COD/N (>1.0) and HRT (>2.0h) are key factors to stabilize PD treating APMW, as well as the presence of biodegradable organic matter. Nevertheless, further studies on the biodegradable content of organic matter and its source are still needed.

In terms of specific activity, the SHDA increased from 14.1 (phase 3) to 22.8 mgN gVSS^–1^ d^–1^, while the SAA increased from 40.8 to 43.4 mgN gVSS^–1^ d^–1^ (Figure 3). This increase aligns with the higher contribution of HD to NRE. Furthermore, the higher rate of COD consumption observed in this phase supports the hypothesis of growth in heterotrophic nondenitrifying microorganisms. Notably, in the SHDA from phase 6, there was a trend toward increasing nitrite accumulation up to 5 mgN-NO_2_^–^ L^–1^, indicating the occurrence of PD. In the SAA, the rate of ammonium removal showed no significant difference between phases 3 and 6. Therefore, the increase in SAA in phase 6 is likely due to the higher nitrite removal rate. Additionally, the N-NO_2_^–^:N-NH_4_^+^ ratio incremented from 1.30 (phase 3) to 1.62 (phase 6), deviating from the theoretical stoichiometric ratio expected for the anammox reaction. These results suggest the occurrence of endogenous heterotrophic denitritation, defined as the reduction of nitrite to nitrogen gas by heterotrophic bacteria using internally stored carbon sources (e.g., polyhydroxyalkanoates or glycogen) rather than external organic matter.^49^ This mechanism may explain the higher SAA observed despite the lower ARE.

It is worth noting that no detectable N_2_O production was observed throughout the entire experimental phases, highlighting an advantage of the PD and anammox process over other anammox-based methods.^2^ Overall, the strategy of gradually transitioning oxidized N-compounds resulted in improved performance, with PD identified as the limiting step in PDA treatment of APMW, at low COD/N ratio and presence of refractory organic matter.

### 3.5 Microbial Diversity Analysis

Microbial community analyses were performed in phases 3 (P3) and 6 (P6), which corresponded to stable operational periods characterized by distinct dominant nitrogen removal pathways. Phase 3 was associated with a higher contribution of anammox to total nitrogen removal, whereas Phase 6 reflected a stabilized PDA regime. This approach allowed a clearer interpretation of microbial shifts linked to process performance rather than transient recovery dynamics.

A total of 39,833 and 45,219 gene sequences were identified in P3 (phase 3) and P6 (phase 6), respectively, suggesting an increase in community richness following the application of APMW. This finding is supported by the Chao1 index, which rose slightly from 104 (P3) to 119 (P6). The community diversity, quantified by the Shannon index, remained stable at 2.6 in both samples. Compared to data previously reported for the inoculum sample, where Chao1 was 48 and Shannon index was 2.2 indexes,^11^ both indexes significantly increased throughout the experimental period, indicating greater community richness and diversity.

Planctomycetota (38.64–11.24%) and Proteobacteria (35.69–79.38%) were found to be the most dominant phyla in both P3 and P6 (Figure 4). These phyla are commonly identified in anammox systems,^8^ and contain AnAOB and DNB microorganisms, respectively. The Proteobacteria phylum plays a crucial role in the denitrification process, interacting with AnAOB, and tends to increase in abundance at higher C/N ratios.^42,43^ In phase 6, coupling PD with anammox led to changes in the relative abundances of both phyla, suggesting an imbalance between AnAOB and DNB, with significant growth of DNB. However, increased AnAOB activity points out to successful PDA establishment.

**Figure 4:**
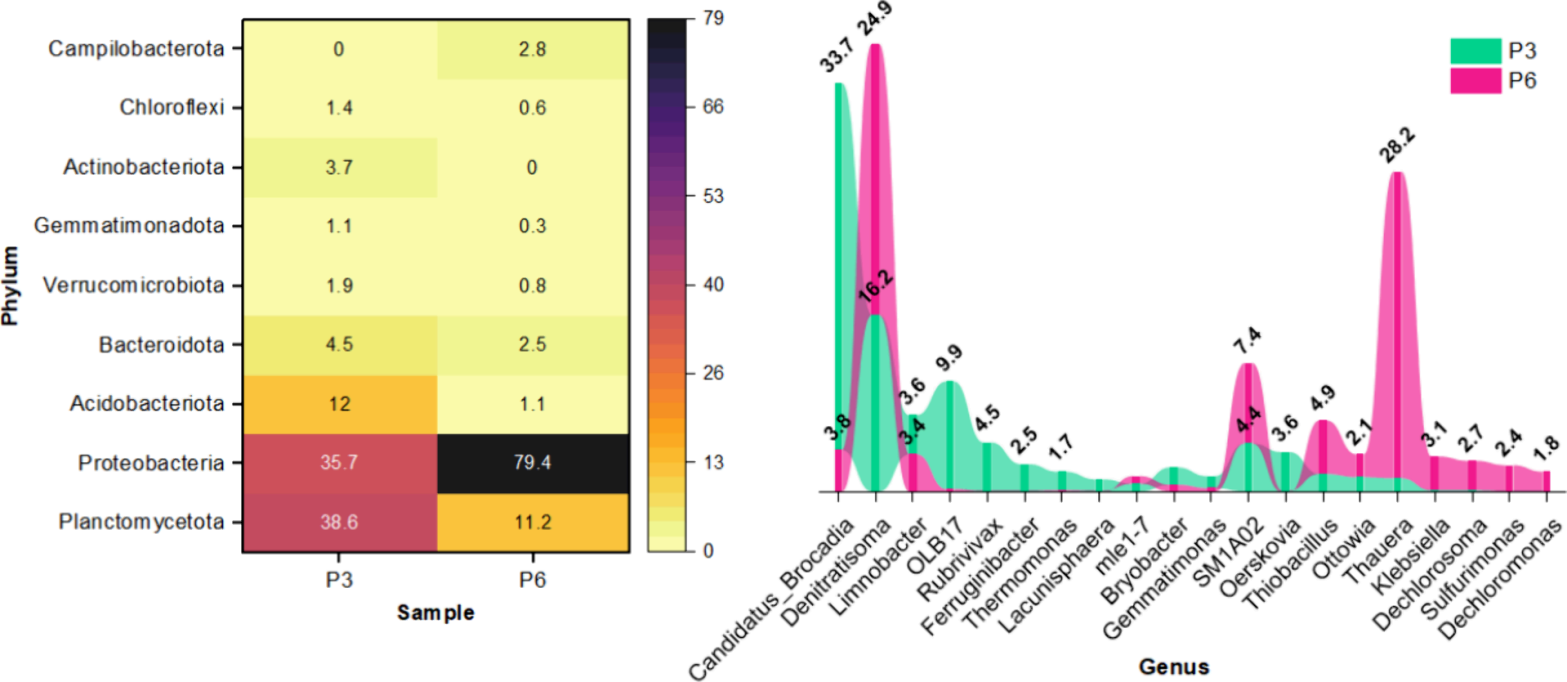
Most abundant microorganisms at phylum (>1.0%) and genus level (>1.5%).

At the genus level within the Planctomycetota phylum, *Candidatus Brocadia* (AnAOB) and *SM1A02* (putative AnAOB^11^;) were identified as responsible for the anammox reaction. Despite the functional versatility of *Ca. Brocadia*, including mixoautotrophic activity under environmental stress conditions,^50^ its relative abundance decreased from 37.76% (I)^11^ to 33.69% (P3) when APMW was introduced to the reactor (Figure 4). Transitioning further to PDA resulted in its lowest abundance at 3.82% (P6), while *SM1A02* increased to 7.42%. Nevertheless, relative anammox abundances as low as 2.5% are expected to sustain dominant anammox activity in PDA reactors.^45^

The *OLB17* genus increased to 9.88% in P3 (Figure 4), possibly in response to the change in wastewater type from SM to APMW. The specific role of this bacterium remains unclear, although it is reported to interact positively with *Ca. Brocadia.* ^51^ Introducing influent organic matter also resulted in an increase in the DNB *Rubrivivax* (4.48%), which was subsequently eliminated during PDA (P6). This reduction may be attributed to its lower affinity for organic compounds compared to other DNBs such as *Denitratisoma, Klebsiella* and *Ottowia,* ^8^ or it may preferentially reduce N-NO_2_^–^ over N-NO_3_^–^. While *Limnobacter* (3.43%), *Klebsiella* (3.05%), *Ottowia* (2.07%) and *Dechloromonas* (1.84%) are complete DNB,^52,53^ *Denitratisoma* (24.88%) and *Thauera* (28.16%) are known by their preference for reducing N-NO_3_^–^ to N-NO_2_^–^ without further reduction.^6^ The proliferation of microorganisms capable of PD correlates with the reactor performance and the specific sludge activity involved in ammonium removal and nitrite accumulation, respectively. In light of this, the prevalence of PD microorganisms might be correlated with the influent N-NO_*x*_^–^ supply. Specifically, the concurrent provision of nitrite and nitrate led to the predominance of complete over partial DNB, while the abrupt shift to nitrate supply promoted the proliferation of partial DNB, at low COD/N ratio.

Typical APMW contains reduced sulfur compounds such as S^2–^, ranging between 5 and 22 mg L^–1^.^7^ Consequently, microorganisms capable of utilizing these compounds may thrive in anammox-based reactors treating this type of wastewater, including genera like *Thiobacillus* and *Sulfurimonas.* ^54^ Both genera are known for their ability to perform complete denitrification and showed increased abundance in phase 6, up to 4.87 and 2.41%, respectively (Figure 4). According to Deng et al.,^54^ sulfur-oxidizing bacteria exhibit growth rates comparable to those of AnAOB, facilitating their stable coexistence in coculture with these microorganisms. Furthermore, as previously described, the proliferation of partial DNB in phase 6 most likely favored AnAOB activity by providing nitrite, allowing the coexistence of diverse microorganisms.

### 3.6 Perspectives for PDA Process toward Efficient Mainstream Anammox

The PDA process is considered a sustainable alternative for nitrogen removal. However, appropriate carbon source selection to attend HD demand and proper nitrate supply remains a challenge.^2^ Organic matter in municipal wastewater can potentially serve as a cost-effective electron donor, reducing the need for an external carbon source. However, most biodegradable fraction of municipal wastewater is removed during pretreatment,^7^ causing the remaining wastewater to be only partially available for DNB. The carbon source recalcitrance negatively effects the NRE by lowering carbon utilization rate and potentially inhibiting microbial activity.^10^

The feasibility and performance of PDA in a single-phase for actual low-strength municipal wastewater treatment have been poorly documented.^55^ As observed in this study, one of the challenges is maintaining a balanced consortium of DNB and AnAOB microorganisms. To address this issue, strategies to enrich microorganisms with a higher affinity for the partial reduction of nitrate to nitrite can be adopted. Studies using acetate,^56^ glycerol,^57^ and formate^58^ as electron donors have indicated improvements in microbial stability. Particularly, acetate has been indicated as an optimal carbon source for enriching such microorganisms and it holds potential for effectively starting up the PDA process. However, the use of external chemical supplies can economically hinder the implementation of PDA. As an alternative, deviating a portion of the raw effluent could provide sufficient biodegradable organic matter for enhanced PD. This approach would become viable once a balanced community of AnAOB and DNB was established. Future research on mainstream PDA process should prioritize the investigation of microbial interactions and metabolic pathways through metagenomic and metatranscriptomic analysis to gain insight into the microbial communities’ responses to varying degrees of organic matter complexity.

## 4 Conclusions

This study successfully demonstrated the domestication of anammox bacteria from synthetic to mainstream actual wastewater, achieving high NRE (94.3%) and ARE (95.2%), showcasing the effectiveness of gradual adaptation. The operational strategy of gradual transition to PDA demonstrated a shift in nitrogen removal dynamics, with HD contributing up to 57.4% of nitrogen removal and complete denitrification taking place. Conversely, the abrupt change from anammox to PDA demonstrated increased partial over complete denitrification pathway, highlighting this strategy as a feasible approach for PDA implementation. Additionally, the complexity of organic matter in actual wastewater is emphasized as a pivotal factor for enhanced nitrogen removal, with refractory organic compounds constraining the PD step.

## Data Availability

Sequence data are available at the National Center for Biotechnology Information (NCBI – http://www.ncbi.nlm.nih.gov) under project accession number PRJNA1135914.

## Notes

All the authors mutually consent to the publication of this manuscript and state it has not been previously published in another journal.

## Abbreviations

AMX: anammox process
AnAOB: anammox bacteria
APMW: anaerobically pretreated municipal wastewater
ARE: ammonium removal efficiency
COD: chemical oxygen demand
CODRE: chemical oxygen demand removal efficiency
DNB: denitrifying bacteria
DO: dissolved oxygen
HD: heterotrophic denitrification
HRT: hydraulic retention time
NRE: nitrogen removal efficiency
PD: partial denitrification
PDA: partial denitrification-anammox
SHDA: specific heterotrophic denitrification activities
NCBI: National Center for Biotechnology Information
N-NH_4_^+^: ammonium
N-NO_3_^–^: nitrate
N-NO_2_^–^: nitrite
N_2_O: nitrous oxide
SAA: specific anammox activities
SM: synthetic medium
VSS: volatile suspended solids
TSS: total suspended solids
